# The 20 year evolution of dobutamine stress cardiovascular magnetic resonance

**DOI:** 10.1186/1532-429X-12-59

**Published:** 2010-10-26

**Authors:** Charaslak Charoenpanichkit, W Gregory Hundley

**Affiliations:** 1 Department of Internal Medicine Section on Cardiology, Wake Forest University School of Medicine, Winston-Salem, North Carolina, USA; 2 Department of Radiology, Wake Forest University School of Medicine, Winston-Salem, North Carolina, USA

## Abstract

Over the past 20 years, investigators world-wide have developed and utilized dobutamine magnetic resonance stress testing procedures for the purpose of identifying ischemia, viability, and cardiac prognosis. This article traces these developments and reviews the data utilized to substantiate this relatively new noninvasive imaging procedure.

## Introduction

The identification of myocardial ischemia and viability is critical to the management of patients with coronary artery disease (CAD), and as such, over the last 20 years, dobutamine stress cardiovascular magnetic resonance (CMR) has been developed for the purpose of identifying these conditions. Today, dobutamine CMR incorporates assessments of left ventricular (LV) wall motion, myocardial perfusion, and tissue characterization. These data are useful for diagnosing and forecasting prognosis in individuals with CAD. In this article, we review the performance of dobutamine stress CMR and describe the clinical utility of this technique for managing patients with known or suspected CAD. In addition, recent innovations are described that may extend principles learned from dobutamine stress CMR into exercise stress CMR.

## Pharmacological effects of Dobutamine and Atropine

**Figure 1:**
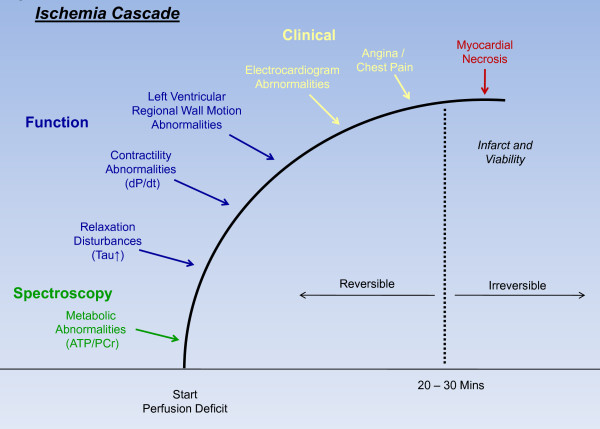
**Sequence of events after coronary artery occlusion**. Ischemic cascade represents the sequence of pathophysiological events following disruption of coronary artery blood flow.

Dobutamine is a synthetic, primarily β1-adrenergic, catecholamine with mild α1- and β2-receptor agonist activity [1,2]. At low doses (≤ 10 μg/kg/min), dobutamine augments myocardial contractility and promotes coronary vasodilation [3]; at higher doses (20-40 μg/kg/min), it causes systematic vasodilation and serves as a positive chronotrope. These later effects increase heart rate and raise myocardial oxygen consumption by increasing myocardial work [4]. Dobutamine can be used during pharmacologic stress testing to identify inducible ischemia due to flow limiting epicardial coronary artery stenosis (Figure 1). Visually, this results in a LV perfusion or wall motion abnormality (WMA).

Since dobutamine increases myocardial oxygen demand, it is advantageous in patients who may need a cardiac stress test but cannot exercise due to peripheral vascular disease, physical incapacitation, or chronic deconditioning [5]. To identify myocardial ischemia during dobutamine stress CMR, it is important to achieve > 85% of the patient's maximum predicted heart rate response (MPHRR) for age during the exam. At this "target" heart rate response, the sensitivity for identifying inducible ischemia using this form of testing increases [6]. To this end, often in conjunction with dobutamine, atropine, a natural alkaloid of "atropa belladonna" and a competitive antagonist of muscarinic cholinergic receptors, is implemented to increase the heart rate by inhibiting the vagal tone.

## Safety Profile for Dobutamine/Atropine Stress

Multiple large studies have examined the safety profile of dobutamine/atropine stress, dobutamine stress echocardiography (DSE) [7-9], and CMR [10-12]. These studies were classified as having major or minor complications. Major complications included severe symptomatic hypotension, acute myocardial infarction (MI), sustained ventricular tachycardia, ventricular fibrillation, rupture of the free wall of the left ventricle or LV septal defect, transient ischemic attack, and death. Minor complications included anxiety, nausea, and atropine poisoning with hallucinations lasting several hours in the absence of myocardial ischemia and hypotension.

In these single and multicenter studies, major adverse events with DSE have been reported in the range of 1 per 2000 to 5 per 1000 [7-9]. Overall, data suggest the rates of major adverse events during DCMR are similar (approximately 1 per 1000) to the rates observed during DSE [10-12]. (Table 1).

**Table 1:** Major and Minor Complications of Dobutamine-Atropine Stress

Study	Modality	Number of Patients	Minor events	Major events	Deaths
Rodríguez García et al (7)	DSE + A	325	57%	21%	1
Picano et al (8)	DSE + A	2799	78%	5%	0
Geleijnse et al (9)	DSE + A	2246	71%	5%	0
Wahl et al (10)	DCMR + A (54%)	1000	64%	6%	0
Kuijpers et al (11)	DCMR	400	71%	3%	0
Hamilton et al (12)	DCMR + A (27%)	469	67%	0	0

A = atropine, DCMR = dobutamine cardiovascular magnetic resonance, DSE = dobutamine stress echocardiography					

Although serious complications are not common, special safety measures are required when performing CMR stress tests. Recommendations for procedures and facilities to perform stress have been previously published [10-14]. Physicians and health-care providers should recognize the importance of identifying ischemia promptly, as major events are usually associated with the continued administration of pharmacological stress in the setting of myocardial ischemia.

## CMR Protocol

Interpretation of LV myocardial perfusion or wall motion is performed during dobutamine/atropine pharmacologic infusions to identify myocardial ischemia and viability. To this end, when assessing LV wall motion, a 17-segment model defined by the American Heart Association (AHA) and American College of Cardiology (ACC) is used in which each segment is scored as: 1 = normal, 2 = hypokinetic, 3 = akinetic, and 4 = dyskinetic. Ischemia is defined as ≥ 1 segments showing inducible WMAs (i.e. an increase in LV wall motion score ≥ 1 during testing; in addition, a biphasic response is considered to indicate ischemia) [15,16]. Once ischemia is detected, tests are terminated; other reasons to terminate testing include symptomatic hypo- or hypertension, marked ventricular arrhythmia, or unexpected neurological findings [10-12,14].

Cardiovascular magnetic resonance imaging is well suited for visualizing LV wall motion during dobutamine stress. With CMR, one can visualize the LV myocardium in multiple tomographic planes [17-19]. The image acquisition can be standardized, thus limiting reliance on the technique of an individual technologist for acquiring high-quality images [20,21]. Dobutamine stress CMR tests are usually performed on short, 1.5 Tesla (T), closed-bore systems, with bore diameters ranging from 55-70 cm (Table 2).

**Table 2:** Utility of dobutamine wall motion stress CMR for identifying ≥ 50% coronary arterial luminal narrowing

Author [Ref#]	Patients (n)	Men (%)	Mean age (years)	Dobutamine dose (μg/kg/min)	Sensitivity (%)	Specificity (%)
Gebker R et al.[47]	455	65	64	40+atropine	91	70

Hundley et al.[41]	163	56	NS	40+atropine	83	83

Korosglou et al.[32]	1493	74	65	40+atropine	89	94

Kuijpers et al.[31]	194	67	62	40	96	95

Nagel et al.[40]	208	71	60	40+atropine	86	86

Paetsch et al.[27]	79	66	61	40+atropine	89	80

Paetsch et al.[45]	150	83	61	40+atropine	78	88

Pennell et al.[24]	25	74	52	20	91	100

Schalla et al.[28]	22	80	60	40+atropine	81	83

Van Rugge et al.[38]	45	82	61	20	81	100

Van Rugge et al.[39]	39	86	60	20	91	80

Wahl et al.[42]	160	-	59	40+atropine	89	84

The performance of dobutamine stress testing with CMR is straightforward. Once contraindications to CMR scanning are confirmed (Table 3), a 12-lead electrocardiogram (ECG) is performed outside of the magnet. Then, after establishing intravenous access, the patient is positioned supine on the MR scanning table. Attached to the patient is a phase array surface coil, ECG monitoring leads, respiratory gaiting belt, pulse oximetry monitor, and brachial blood pressure cuff [22]. A registered nurse (to administer medications and record rhythms) and physician continuously monitor the heart rate and rhythm, blood pressure, oxygen saturation, and respiratory rate throughout the study [14,22].

**Table 3:** Contraindication for dobutamine/atropine stress CMR

Dobutamine	• Severe arterial hypertension (≥ 220/120 mmHg)
	• Unstable angina pectoris
	• Significant aortic stenosis (aortic valve gradient > 50 mmHg or aortic valve area < 1 cm2)
	• Complex cardiac arrhythmias including uncontrolled atrial fibrillation
	• Hypertrophic obstructive cardiomyopathy
	• Myocarditis, endocarditis, pericarditis
	• Uncontrolled congestive heart failure
	• Previous manifestations of hypersensitivity to dobutamine
**Atropine**	• Narrow angle glaucoma, myasthenia gravis, obstructive uropathy, obstructive gastrointestinal disorders

**CMR- examination**	• Non compatible biometallic implants and pacemaker or implanted defibrillators (ICDs)
	• Claustrophobia

**Figure 2:**
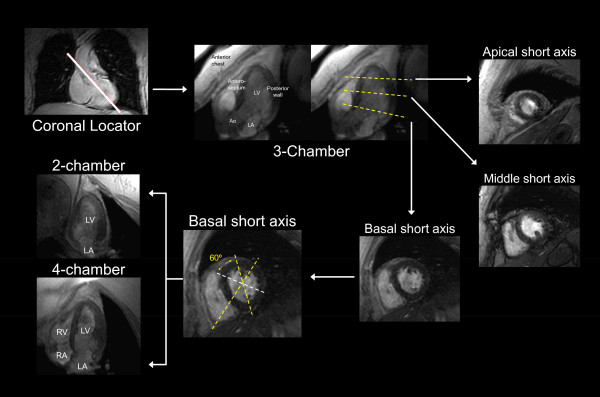
**(Panel A): Myocardial segmentation during dobutamine stress CMR**. Panel A: Strategy for obtaining 3 apical (3-chamber, 4-chamber, and 2-chamber) and 3 short-axis (basal, middle, and apical) cardiovascular magnetic resonance views of the left ventricle. On each image the myocardium is gray and the blood pool white. The white solid line on the coronal locator, and the white dotted lines on the 3-chamber view and basal short-axis view indicate the slice positions for obtaining the subsequent views demarcated by the white arrows.

**Figure 3:**
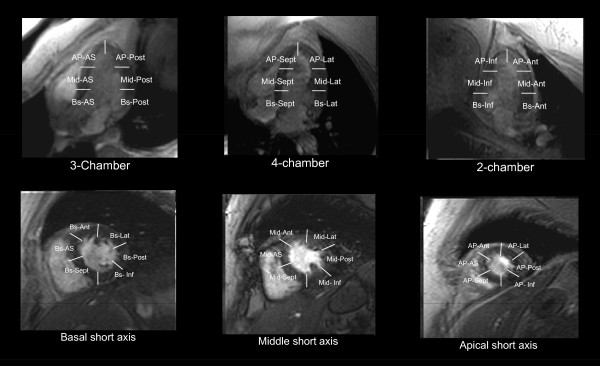
**(Panel B): Myocardial segmentation during dobutamine stress CMR**. Panel B: White lines demarcate the apical (AP), middle (Mid), and basal (Bs) segments of the anteroseptal (AS), anterior (Ant), lateral (Lat), posterior (Post), and inferior (Inf) walls.

At baseline in dobutamine stress CMR, cine white blood images are obtained in three apical views: 2, 3, and 4-chamber views (Figures 2 and 3), and in at least three short-axis planes (the base, middle portion and apex). In situations where LV volumes are to be acquired, it is recommended to also acquire short-axis slices (spanning from the LV base to the apex) in order to calculate LV volumes using a Simpson's rule technique [23].

Images are acquired during five-minute dobutamine infusions of 7.5 mcg/kg/min, followed by 20-50 μg • kg^-1 ^• min^-1 ^designed to achieve 85% of the MPHRR for age. If 85% MPHRR is not achieved during obutamine stress CMR, atropine is administered in 0.1 to 0.3-mg increments to augment the heart-rate response. At each stress level, 3 cine short- and 3 cine- long-axis images of the LV are acquired and images are compared side by side with baseline images for assessment of the development of regional wall motion abnormalities, which indicate stress induced ischemia. Criteria for terminating pharmacologic stress are shown in Table 4. At the completion of the study, additional images are obtained to confirm that LV wall motion has returned to baseline.

**Table 4:** Criteria for stopping dobutamine infusion

Criteria	
**1**	Target heart rate achieved

**2**	Intractable symptoms: severe angina pectoris, severe dyspnea

**3**	New or worsening WMA in ≥ 1 LV segment

**4**	Drop in systolic blood pressure of ≥ 40 mmHg with change in reported symptoms

**5**	Blood pressure increase greater than 240/120 mmHg

**6**	Complex cardiac arrhythmias

**7**	Patient request

## CMR techniques for dobutamine stress CMR

From 1990-96, spoiled gradient-echo imaging techniques were used to identify LV regional WMA induction of ischemia during dobutamine stress CMR [24]. However, in the setting of impaired global or regional LV systolic function that promotes slow intracavitary flow, steady-state free precession (SSFP) imaging has been utilized. This type of imaging sequence yields a higher blood pool signal intensity and thus a very high contrast to noise ratio between intracavitary blood and the myocardium. In combination with parallel image acquisitions (SENSE, sensitivity encoding), one may acquire up to 50 phases/cardiac cycle during an end-expiratory breathhold of about 6 seconds at heart rates of 190-220 bpm [25-27]. The in-plane spatial resolution of SSFP cine scans usually lies in the range of 1.6 mm × 1.6 mm with a slice thickness of 8 mm.

Real time imaging techniques that permit accurate cardiac functional studies without ECG triggering or breath-holding have been implemented during dobutamine stress CMR in patients with cardiac arrhythmia (frequent ectopic ventricular beats and atrial fibrillation) and those incapable of breath holding. Schalla, *et al*[28] have demonstrated the feasibility and accuracy of real-time MR imaging under stress conditions, however, both the spatial and temporal resolutions were reduced compared with the standard breath-hold SSFP approach. New sequences with parallel imaging in the time domain, such as k-t BLAST (broad-use linear acquisition speed-up technique) and k-t SENSE [29,30] may eventually lead to further improvement in real-time imaging (Table 5).

**Table 5:** Advantages and Disadvantages of White Blood Cine Imaging Techniques

Technique	Advantage	Disadvantage
FGRE (breathhold)	Reliable at elevated regular heart rate	Breathhold = 12 seconds
FGRE (respiration-triggered)	No Breath-holding	45-second scan
SSFP	Apical views	Artifact at elevated heart rates
SENSE	Rapid acquisition	Unknown reliability; potentially low temporal resolution
Real-time	Rapid acquisition	Noise, lower spatial and temporal resolution

Abbreviations: FGRE = fast gradient-echo, SSFP = steady-state free precession, SENSE = sensitivity encoding.

Myocardial tissue tagging has been utilized in two fashions during dobutamine stress CMR to enhance the sensitivity of wall motion results for identifying ischemia due to flow limiting epicardial coronary artery stenoses. In the first, Kuijpers, *et al*.[31] demonstrated the utility of tissue tagging to enhance qualitative identification of LV WMA indicative of ischemia. In the second, quantitative methods have proven useful to identify abnormalities of myocardial strain. In fact, tagged CMR images have been used to quantify LV wall motion in systole and diastole to detect early evidence of ischemia during low dose dobutamine infusions. Such an imaging approach may prove useful in limiting the duration (and potential adverse eventws) of high dose dobutamine stress CMR [32]. In addition, Paetsch, *et al*.[33] has recently reported that diastolic parameters (time to peak untwist) derived from myocardial tagging could identify patients with significant coronary artery stenosis at the level of low-dose dobutamine stress. Importantly however, studies with larger participant numbers as well as reliable quantitative software are needed before these early preliminary results can be adopted clinically [34].

While the majority of dobutamine stress CMR has been performed at 1.5T, studies on 3.0 Tesla systems have been attempted [35]. Current SSFP cine sequences at 3.0 Tesla suffer from susceptibility artifacts/magnetic field inhomogeneities rendering endocardial border recognition difficult particularly during the high flow states encountered at high levels of intravenous dobutamine and atropine. New multitransmit technology may overcome these limitations in the near future [36].

## Clinical applications - Identification of inducible ischemia

**Figure 4:**
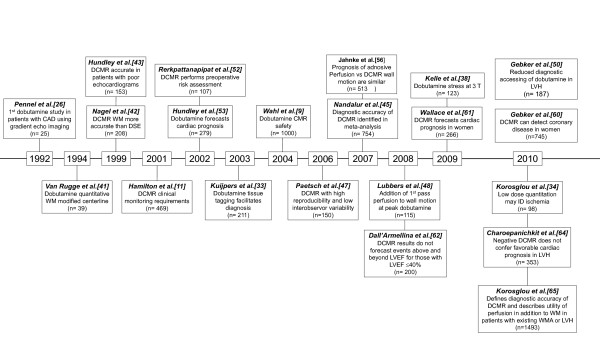
**Timeline displaying the progression of dobutamine stress CMR research over the past 20 years**. Each box represents a published manuscript of peer reviewed original research with the first author, a short summary, and the sample size (n) within the study.

Pennell, *et al*.[24] reported the first use of LV wall motion analyses during dobutamine stress CMR in 25 patients with exertional chest pain. Results from dobutamine stress CMR were compared with thallium-201 single photon emission tomography (SPECT) and contrast coronary angiography (Figure 4). The subjects received dobutamine infusions of up to 20 μg/kg/min and underwent conventional gradient-echo cine dobutamine stress CMR. The sensitivity of dobutamine stress induced LV wall motion abnormalities for detecting SPECT evidence of ischemia was 91%, and there was 90% agreement between SPECT and dobutamine stress CMR for identifying results indicative of ischemia. Twenty-one (96%) of these patients had reversible myocardial ischemia shown by dobutamine thallium tomography, and 20 (91%) had reversible WMAs shown by dobutamine stress CMR. Other early studies confirmed these results by demonstrating the overall sensitivities of 81-84% for detecting coronary arterial luminal narrowings ≥ 50% [37-39].

Limitations of these early studies include the fact that they were relatively small participant numbers (20-60 patients per study), were performed in single centers, and enrolled participants with known CAD prior to stress testing. Also, LV wall motion was analyzed at baseline and compared with peak stress; it was not visualized continuously throughout testing. As a result, the tests were terminated prematurely when patients developed chest pain. Thus, while these studies highlighted the feasibility of dobutamine administration in the CMR environment, the clinical utility of dobutamine stress CMR remained in question.

Two larger prospective studies in the late 1990's established the clinical utility of dobutamine stress CMR. In the first, Nagel, *et al*.[40] compared the results of dobutamine stress CMR with DSE in 208 subjects who underwent both procedures for detection of significant CAD, defined as > 50% coronary arterial luminal diameter stenosis. Dobutamine was infused intravenously up to 40 μg/kg/min in order to achieve ≥ 85% of the MPHRR for age. Dobutamine stress CMR demonstrated superior sensitivity (86% vs 74%), specificity (86% vs 70%), and diagnostic accuracy (86% vs 70%) in comparison with DSE (p < 0.05 for all). These differences were most pronounced in patients with suboptimal acoustic windows.

**Figure 5:**
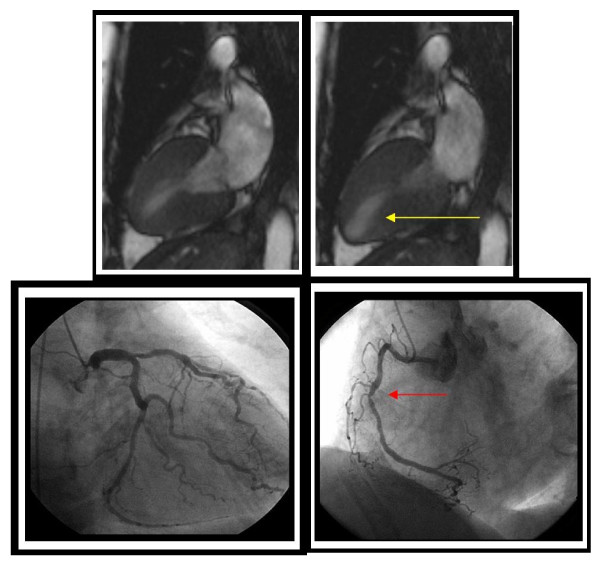
**Inducible left ventricular wall motion abnormalities indicative of ischemia**. A resting end-systolic frame from a 2-chamber view is shown in the top left corner demonstrating normal left ventricular contraction with no wall motion abnormalities. The top right image shows a peak dobutamine end-systolic cine view; the yellow arrow highlights hypokinesis of the inferoapical region. The bottom images are a caudal view of the right anterior oblique projection during contrast coronary angiography. The bottom left image exhibits a 70% proximal left anterior descending, 80% obtuse marginal, and 60% distal circumflex lesions. The red arrow in the bottom right highlights an 80% lesion in the mid-right coronary artery.

In the second study, Hundley, *et al*.[41] performed complimentary work in 153 patients with a non-diagnostic DSE despite the use of second harmonic imaging. When compared with contrast coronary angiography, the sensitivity and specificity of dobutamine stress CMR were both 83% for detecting coronary arterial luminal narrowings of ≥ 50% by contrast coronary angiography (Figure 5). After these studies confirming clinical utility by Nagel, *et al*., and Hundley *et al*., Paetsch, *et al*.,[27] compared the diagnostic value of dobutamine stress MR wall motion analyses with adenosine stress MR wall motion and perfusion analyses in the same study participants during a single session examination. They found dobutamine induced LV wall motion abnormalities accurate and more sensitive and specific for identifying inducible ischemia compared to perfusion assessments alone. An important limitation of this work was the absence of delayed enhancement methods to identify infarcts involving the LV myocardium.

Detection of new or worsening WMAs in the subgroup of CAD patients with resting WMAs is known to be especially difficult. However, Wahl *et al*.[42] evaluated the diagnostic accuracy of dobutamine stress CMR in 160 consecutive patients with pre-existing WMAs who had sustained prior MI and/or prior coronary arterial revascularization. The sensitivity and specificity for detecting coronary arterial luminal narrowing of ≥ 50% were 89% and 84%, respectively. Additionally, the overall sensitivity for detection of significant CAD in patients with single-, double-, and triple-vessel disease was 87%, 91%, and 100%, respectively. This study demonstrated that high dose dobutamine stress CMR can be useful even in patients with preexisting WMAs and history of coronary revascularization. As shown in Table 2, a recent meta-analysis revealed a sensitivity of 0.83 (95% confidence interval 0.79 to 0.88) and specificity of 0.86 (95% confidence interval 0.81 to 0.91) of high dose dobutamine stress CMR for identifying > 50% coronary arterial luminal narrowings in patients with a relatively high disease prevalence (disease prevalence = 70.5%) [43].

Beside its consistently high diagnostic accuracy, dobutamine stress CMR has been reported to provide a low interobserver variability (kappa = 0.81), and a high reproducibility (p = 0.91).[43] In the setting of multiple observers from different institutions performing a diagnostic reading of dobutamine stress CMR examinations acquired from a single center, the interobserver variability was low for identifying inducible LV WMAs indicative of coronary arterial luminal narrowings ≥ 50% [44,45].

As imaging technology has advanced, it is now possible to examine myocardial perfusion in addition to wall motion during dobutamine stress. In 2008, Lubbers, *et al*.[46] and Gebker, *et al*.[47] were the first to assess the additional value of first pass myocardial perfusion imaging during peak dose of dobutamine stress CMR for the detection of myocardial ischemia. The addition of first-pass myocardial perfusion imaging to wall motion assessments during peak-dose dobutamine stress CMR improved sensitivity for the diagnosis of coronary artery disease. Gebker, *et al*., identified that the accuracy of dobutamine stress CMR-wall motion was influenced by LV geometry: in patients with concentric remodeling or hypertrophy, additional first-pass perfusion imaging during high dose dobutamine stress improved the diagnostic accuracy for the detection of CAD [48].

Several small studies have reported on the effectiveness of exercise as a mechanism to induce LV WMA indicative of ischemia. Exercise testing offers important additional information such as exercise capacity, blood pressure response, development of arrhythmias, and the presence of symptoms (e.g. chest pain) during exercise [49]. Jekic, *et al*. examined 20 healthy subjects using a treadmill exercise CMR protocol and found that all left ventricular segments had sufficient visual assessments of wall motion [49]. In addition, Rerkpattanapipat, *et al*., demonstrated a 79% sensitivity of maximal exercise treadmill MRI for identifying ≥ 50% coronary arterial luminal narrowings in patients with primarily single vessel CAD [50].

## Prognostic value of DCMR stress

Several studies, including those involving > 1000 patients, have provided insight into subgroups of patients where dobutamine stress CMR assessment of ischemia may forecast cardiac prognosis. In 2002, Hundley, *et al*.[51] evaluated 279 patients who underwent dobutamine stress CMR followed for an average of 20 months. In a multivariable analysis, the presence of ischemia or an LVEF < 40% were associated with future MI and cardiac death, independent of the presence of risk factors for coronary arteriosclerosis or MI. In a subsequent study of these same participants, inducible ischemia involving the LV myocardial apex was associated with future MI and cardiac death (independent of the location of the myocardial segment within the apex); ischemia isolated to basal and middle segments was not [52].

Subsequent to these studies, Kuijpers, *et al*.,[53] followed 214 patients with a negative dobutamine stress CMR study with an average of 24 months. Dobutamine-CMR showed a positive and negative predictive value of 95% and 93%, respectively, for identifying major adverse cardiac events. In participants without evidence of ischemia the reported cardiovascular event-free survival rate was 96.2%.

In comparison with adenosine stress CMR, results from dobutamine stress CMR have similar prognostic accuracy. In a larger patient population undergoing combined dobutamine stress CMR and adenosine first-pass perfusion MR imaging, Jahnke, *et al*., demonstrated similar 2- year event-free survival rates for both stressors [54]. Most importantly, these authors identified a 2-year relatively event-free period of cardiac events when stress CMR tests were negative. In the future, dobutamine stress CMR results may be useful to accomplish decisions regarding clinical care [54].

CMR also has been utilized to determine preoperative cardiovascular risk in patients undergoing non-cardiac surgery. In the subgroup of patients with intermediate clinical predictors of future cardiac events, a positive dobutamine stress CMR test proved to be an independent factor for predicting MI, cardiac death or congestive heart failure during or after the surgery [50].

## Dobutamine stress CMR in Women

The diagnosis of CAD in women presents challenges not seen in testing men [55,56]. Differences in the epidemiology of CAD between men and women render women at generally lower risk than their male counterparts until the seventh decade of life. The high prevalence of nonobstructive CAD and single-vessel disease in women generally results in decreased diagnostic accuracy and higher false-positive rate for noninvasive testing in women versus men [55,56]. The substantial under representation of women in studies of noninvasive testing further limits the evidence-based information on which to base clinical decision making [57].

To this end, Gebker, *et al*.,[58] performed a comparative study to assess the diagnostic value of dobutamine stress CMR for the detection of CAD in men and women. They reported that the diagnostic value for identifying myocardial ischemia indicative of coronary arterial luminal narrowings of > 70% was similar for men (sensitivity, specificity, accuracy; 86%, 83%, 85%) and women (85%, 86%, 85%, respectively).

Wallace, *et al*.[59] recently determined the utility of dobutamine stress CMR results for predicting cardiac prognosis in women. Two hundred sixty-six consecutively referred women who underwent dobutamine stress CMR were followed up to an average of 6.2 years after dobutamine stress CMR. Similar to men, dobutamine stress CMR results were found efficacious for identifying women at risk for future MI or cardiac death. In women with known or suspected ischemic heart disease, dobutamine stress CMR results were independent predictors of cardiac events after accounting for known risk factors for CAD and MI.

## Limitations of dobutamine stress CMR wall motion analyses

Several studies have identified patient populations in which wall motion assessed during dobutamine stress CMR may be insufficient for identifying those at risk for CV events. The first such situation involves dobutamine stress CMR in patients with a severe reduction in LVEF. Dall'Armellina, *et al*.[60] demonstrated that in individuals with moderate to severe reductions in LVEF (LVEF < 40%), a dobutamine-induced increase in wall motion score index (a marker of inducible ischemia) was not predictive of future MI and cardiac death beyond the assessment of resting LVEF.

Increased LV wall thickness or left ventricular hypertrophy (LVH) appear to offer prognostic implications independent of the presence of dobutamine induced LV WMA. Recently, Walsh and colleagues[61] identified that increased LV end-diastolic wall thickness in the base of the septum or lateral wall was associated with MI and cardiac death in individuals with a resting LVEF > 55% and no inducible LV WMA indicative of ischemia after dobutamine infused to achieve 85% of the MPHRR for age. In a second study, Charoenpanichkit, *et al*.[62] found that LVH was an independent prognostic marker above and beyond assessments of LV WMA. In fact, in those with LVH but without dobutamine induced WMA, the future risk of MI and cardiac death was found similar to those with a preserved LVEF and the presence of LV WMA.

In one of the largest reported dobutamine stress studies worldwide, Korosoglou, *et al*.[63] reported the outcomes of 1493 patients undergoing dobutamine stress. Overall, patients' prognosis and diagnostic accuracy were similar to those observed in prior studies with smaller numbers of participants. In this relatively large, single-center study, dobutamine-induced LV wall motion and perfusion abnormalities predicted future adverse CV events after accounting for established risk factors of CV disease.

The relatively large number of study participants also allowed the investigators to reach important conclusions regarding patient subgroups and associated comorbidities. Important conclusions drawn from this study include: first, the identification of dobutamine-induced wall motion abnormalities forecasted CV prognosis in those with or without myocardial perfusion deficits. The converse, however, was not true, as perfusion assessments only provided incremental prognosis information in individuals who did not experience an inducible wall motion abnormality during intravenous dobutamine. Moreover, the prognostic utility of these additional perfusion images occurred only in those with existing LV wall motion abnormalities at rest, those with known coronary disease, or those with LV hypertrophy.

Second, the presence of dobutamine-induced LV wall motion abnormalities forecasted cardiac prognosis in individuals regardless of the pretest probability of coronary artery disease (low, intermediate, or high). The absence of inducible LV wall motion abnormalities only conferred a favorable CV prognosis in those who were at low or intermediate risk.

**Figure 6:**
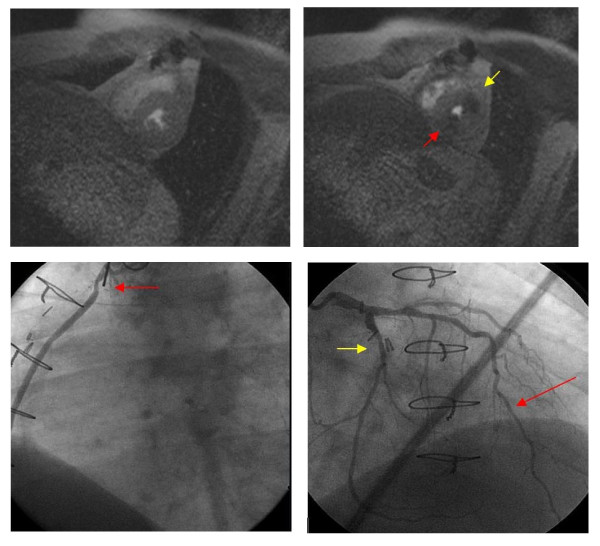
**Utility of Perfusion Testing During Dobutamine Stress: *Top Left Image: *Resting mid-left ventricular short axis with no perfusion abnormalities**. *Top Right Image: *Resting mid-left ventricular short axis slice after dobutamine stress demonstrating perfusion abnormalities in the inferior (red arrow) and anterolateral (yellow arrow) regions. *Bottom Left Image: *Left anterior oblique view of the SVG to distal RCA graft; red arrow points to a 70% lesion in the proximal body of the graft. *Bottom Right Image: *Right anterior oblique cranial view of the left coronary system; red arrow points to diffuse disease in the mid-and distal left anterior descending artery; yellow arrow points to a 60% lesion in the mid-circumflex coronary artery.

Finally, the results (positive or negative for ischemia) of either wall motion or perfusion stress tests did not add incremental information regarding CV prognosis in individuals with a severely reduced LV ejection fraction at rest (Figure 6). The results of this large study suggest one should consider stress CMR imaging strategies according to patient co-morbidities on an individualized basis. In some individuals wall motion analyses may be sufficient; whereas in others, wall motion may need supplementation with perfusion imaging.

## Dobutamine stress CMR for detection of myocardial viability

Commonly, clinically important viable myocardium is defined as tissue that retains contractile capability 6 weeks to 6 months following a successful coronary arterial revascularization procedure. While several CMR imaging and spectroscopic methods exist to identify myocardial viability, 3 are used in the clinical setting: measurement of resting LV end-diastolic wall thickness, usual interpretation or quantitation of LV systolic or diastolic performance during low dose (5 to 10 mcg/kg/min) dobutamine stress CMR or quantitation of non viable scarred or infracted myocardial tissue using late gadolinium enhancement imaging. In this latter situation, as would be expected, an inverse relationship exists between the amount of scarred tissue and the chance a myocardial segment will recover systolic thickening after successful coronary arterial revascularization. For purposes of this review, presented material will focus primarily on the association between dobutamine induced changes in LV performance and future resting LV performance after successful coronary artery revascularization.

The first report of using low dose dobutamine infusions to identify myocardial segments in patients with coronary arteriosclerosis that would respond to coronary artery revascularization was by Cigaroa, *et al*., in 1990 using DSE [64]. In this study, an improvement in LV wall motion score index [WMSI] (an assessment of segmental wall motion/the number of LV myocardial segments assessed) during dobutamine predicted LV WMSI after coronary artery revascularization. Since LV wall motion can be difficult to assess in individuals with poor quality transthoracic echocardiograms, other investigators have sought to determine the utility of CMR for identifying contractile reserve indicative of future recovery of function after successful coronary artery revasculazation.

Baer, *et al*.[65] were one of the first investigative groups to report that implementation of low dose dobutamine stress during CMR could be used to measure LV myocardial thickening during low dose dobutamine. A LV wall thickening response of < 1 mm denoted segments that lacked viability. They concluded that dobutamine stress CMR results were better predictors of residual metabolic activity as opposed to myocardial SPECT imaging with a sensitivity of 81% versus 72% and a specificity of 95% vs. 89%, respectively. In another study from this same group, a CMR-derived systolic wall thickening of ≥ 2 mm during low dose dobutamine was found to predict LV segmental functional recovery 4-6 months after coronary arterial revascularization [66]. Similar results were reported by Sandstede, *et al*.[67]: the sensitivity and specificity of low dose dobutamine for the prediction of myocardial viability were 76% and 100%, respectively for patient-related analyses based on coronary artery distribution.

Myocardial tissue tagging has been used as an adjunct to dobutamine stress CMR to predict the viability of hibernating or stunned myocardium. Geskin, *et al*.[68] demonstrated that an improvement in contractility within the midwall and subepicardium predicted future functional recovery of the LV myocardium after coronary arterial revascularization. Similar results have been found by Bogaert, *et al*.[69] in studies of patients with single vessel disease one week after successful reperfusion of a first transmural anterior MI. Sayad, *et al*.[70] found that the LV end systolic wall thickness measured during low dose dobutamine infusion in patients with chronic ischemic heart disease correlated with the LV end systolic wall thickness 6 months after coronary arterial revascularization.

Dendale and colleagues[71] evaluated the feasibility of stress CMR use for the detection of viability after acute MI. Gradient echo MR images were analyzed for WMA during low doses of dobutamine in 37 patients sustaining a recent MI. The authors concluded that low-dose dobutamine MR imaging is a safe alternative to echocardiography to predict recovery of WMA after MI. In a quantitative analysis, Saito and colleagues[72] compared dobutamine stress CMR with DSE for identifying myocardial viability in subjects with LV dysfunction at rest. The sensitivity of dobutamine stress CMR with tagging was noted to be 76% whereas that of DSE was 66%. The specificity of dobutamine stress CMR was 86% and that of DSE was 100%. The accuracy of dobutamine stress CMR was 78% while that of DSE was 72%.

In a recent study, Kramer, *et al*.[73] found that both dobutamine stress CMR and DSE are sensitive and accurate techniques for predicting functional improvement after reperfused MI. In this study, echocardiography was used as the reference for determination of functional recovery. Interestingly, the results of MR tagging analyses demonstrated more segments that would exhibit functional recovery at follow-up when compared with echocardiography. They also demonstrated that dobutamine stress CMR within 3 days of an acute MI was safe and well tolerated, even in patients who underwent coronary artery stenting. Another study by Zamorano, *et al*.[74] compared thallium-201 redistribution imaging, cine dobutamine stress CMR, and DSE for the assessment of myocardial viability in 10 ischemic patients scheduled for heart transplantation. The explanted hearts were analyzed to quantify fibrosis using Mason trichrome staining. The authors noted that the highest agreement was found between DSE and CMR. In this study, perfusion imaging by thallium-201 scintigraphy was found to be more sensitive but less specific than assessment of contractile reserve for the detection of myocardial viability.

**Figure 7:**
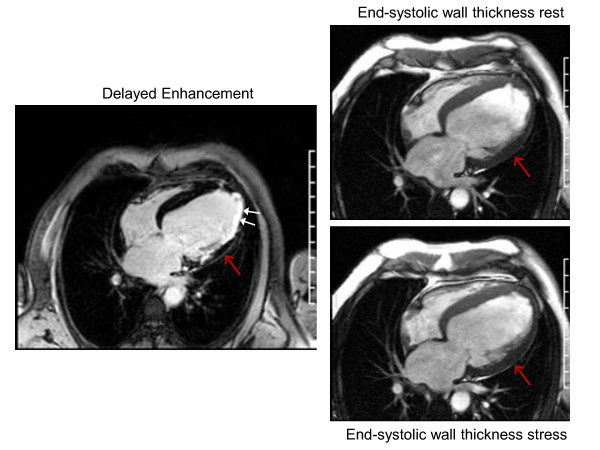
**Case example of contractile reserve supplementary delayed enhancement imaging for the purpose of identifying myocardial viability**. In the left panel, white arrows demarcate extensive delayed enhancement suggesting indicating marked fibrosis. The red arrow, however, denotes a region with < 50% delayed enhancement. The red arrows in the right panels (top = rest and bottom = stress) demarcate no improvement in end-systolic wall thickness with low-dose dobutamine stress. This finding suggests this segment will not improve wall thickening after coronary arterial revascularization.

A major strength of CMR in general relates to the flexibility of the technology such that CMR functional measures of contractile reserve can be acquired with LGE (infarct detection) in a single comprehensive exam (Figure 7). The complementary nature of these modalities may lead to improved detection of myocardial viability. As shown by Kramer, *et al*.[75] in a group of patients with reperfused MI, using this combined technology, these investigators visualized infarcted or scarred myocardium and simultaneously assessed contractile reserve in residual myocardium.

In several other single-center studies, investigators have performed dobutamine stress CMR both wall motion studies and late gadolinium enhancement imaging to predict improvement of LV systolic function after coronary arterial revascularization. These studies have been performed in patients sustaining acute myocardial injury or experiencing chronic ischemic heart disease [76-79]. Lauerma, *et al*.,[76] and Wellnhoffer, *et al*.,[77] identified that increased thickening during dobutamine stress CMR was very useful for predicting functional recovery of the LV myocardium in patients with an intermediate amount of late gadolinium enhancement (1% to 49% of the transmural thickness of an individual myocardial segment). Bove, *et al*.,[78] demonstrated that in segments with 1 to 50% infarct transmurality, a normal dobutamine response helped to differentiate segments with greater functional recovery after revascularization. Motoyasu, *et al*.,[79] demonstrated similar results in 23 patients with acute reperfused MI. Thus, in both acute and chronic ischemic heart disease, it is those segments with intermediate (≥ 1% but < 50%) transmural enhancement where low dose dobutamine stress CMR results exhibit additional clinical accuracy for predicting an ability of a myocardial segment to recover function after coronary arterial revascularization.

Some authors have suggested that combination of scar determination and contractile reserve during dobutamine administration improves the overall accuracy of CMR for determining myocardial viability. Kaandorp, *et al*.,[80] tested this hypothesis in 48 patients with ischemic cardiomyopathy and found that 61% of segments with an intermediate extent of scar tissue on CMR had contractile reserve and 39% lack contractile reserve. As new treatment strategies develop for managing ischemic cardiomyopathy, such as, stem cell placement, specialized left ventricular assist device insertion, or multi-lead pacing systems, the combination of tissue characterization and low dose functional stimulation studies may provide the data necessary to optimally deliver these specialized therapies. Since CMR does not utilize ionizing radiation, it may become one of the preferred testing modalities to longitudinally gather this information in order to optimally direct delivery of these specialized therapies.

In addition to device related and other interventional treatment strategies, low dose dobutamine CMR wall motion responses may be able to guide medication administration or forecast cardiac prognosis. A single study has shown that an increase of LVEF during low-dose dobutamine stress testing also predicts the improvement of LV function after initiation of β-blocker therapy [81].

**Figure 8:**
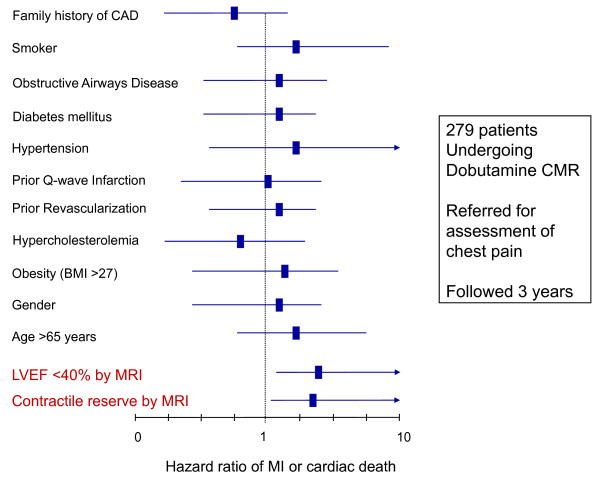
**Multivariable analysis for contractile reserve predicting cardiac events**. Hazard ratios ± 95% confidence intervals (x-axis) for myocardial infarction or cardiac death in a multivariable (y-axis) analysis. As shown, contractile reserve is a predictor of adverse cardiac events after accounting for risk factors for cardiac events.

Dobutamine assessments of contractile reserve appear to predict cardiac events. In a study of Hundley, *et al*., subgroup analyses found the presence of contractile reserve at low doses of dobutamine (7.5 to 10 mcg/kg/min) in patients with coronary artery disease predicted future MI and cardiac death after accounting for other risk factors for cardiovascular events (Figure 8) [51]. Thus, in addition to selecting patients that would improve LV performance after successful coronary artery revascularization; low dose dobutamine CMR results also forecast individuals at high risk for future MI and cardiac death.

## Summary

Dobutamine stress CMR is useful technique for identifying myocardial ischemia and viability with high diagnostic accuracy. This technique is of particular utility in patients not well suited for other noninvasive imaging techniques. Beside its diagnostic value, dobutamine stress CMR results provide relevant prognostic information regarding future risk of MI and cardiac death. Performance of dobutamine stress CMR requires appropriately trained staff, monitoring and safety measures. The combined assessment of wall motion, perfusion and scar transmurality during single session CMR imaging may prove to be imaging method of choice to guide to the clinical management of patients with known or suspected coronary artery disease.

## Abbreviations

**ACC**: American College of Cardiology; **AHA**: American Heart Association; **CAD**: coronary artery disease; **DCMR**: dobutamine stress cardiovascular magnetic resonance; **DSE**: dobutamine stress echocardiography; **ECG**: electrocardiogram; **LGE**: late gadolinium enhancement; **LV**: left ventricular; **LVEF**: left ventricular ejection fraction; **LVH**: left ventricular hypertrophy; **MI**: myocardial infarction; **MPHRR**: maximum predicted heart rate response for age; **PET**: positron emission tomography; **SENSE**: sensitivity encoding; **SSFP**: steady-state free procession; **T**: tesla; **WMA**: wall motion abnormalities.

## Competing interests

The authors declare that they have no competing interests.

## Authors' contributions

Each author participated in researching the relevant articles and drafting and revising the manuscript. All authors have read and approved the final manuscript.

## References

[B1] Ruffolo RR Jr The pharmacology of dobutamine Am J Med Sci 1987 294 4 244 8 10.1097/00000441-198710000-00005 3310640

[B2] Vallet B Dupuis B Chopin C Dobutamine: Mechanisms of action and use in acute cardiovascular pathology Ann Cardiol Angeiol (Paris) 1991 40 6 397 402 1859148

[B3] Kobori M Shida K Negishi H Masuda Y Hosoyamada A Evaluation of dopamine and dobutamine for use in circulatory depression associated with induced total spinal block Masui 1991 40 2 190 201 2020094

[B4] Iskandrian AS Verani MS Heo J Pharmacologic stress testing: Mechanism of action, hemodynamic responses, and results in detection of coronary artery disease J Nucl Cardiol 1994 1 1 94 111 10.1007/BF02940016 9420675

[B5] Dagianti A Penco M Agati L Sciomer S Dagianti A Rosanio S Fedele F Stress echocardiography: Comparison of exercise, dipyridamole and dobutamine in detecting and predicting the extent of coronary artery disease J Am Coll Cardiol 1995 26 1 18 25 10.1016/0735-1097(95)00121-F 7797748

[B6] Lewandowski TJ Armstrong WF Bach DS Reduced test time by early identification of patients requiring atropine during dobutamine stress echocardiography J Am Soc Echocardiogr 1998 11 3 236 42 10.1016/S0894-7317(98)70085-9 9560747

[B7] Rodriguez Garcia MA Iglesias-Garriz I Corral FF Garrote CC Alonso-Orcajo N Branco L Picano E Evaluation of the safety of stress echocardiography in Spain and Portugal Rev Esp Cardiol 2001 54 8 941 8 11481108 10.1016/s0300-8932(01)76429-9

[B8] Picano E Mathias W Jr Pingitore A Bigi R Previtali M Safety and tolerability of dobutamine-atropine stress echocardiography: a prospective, multicentre study. Echo Dobuta, ime International Cooperative Study Group Lancet 1994 344 8931 1190 2 10.1016/S0140-6736(94)90508-8 7934540

[B9] Geleijnse E Fioretti PM Roelandt JR Methodology, feasibility, safety and diagnostic accuracy of dobutamine stress echocardiography J Am Coll Cardiol 1997 30 3 595 606 10.1016/S0735-1097(97)00206-4 9283514

[B10] Wahl A Paetsch I Gollesch A Roethemeyer S Foell D Gebker R Langreck H Klein C Fleck E Nagel E Safety and feasibility of high-dose dobutamine-atropine stress cardiovascular magnetic resonance for diagnosis of myocardial ischemia: Experience in 1000 consecutive cases Eur Heart J 2004 25 14 1230 6 10.1016/j.ehj.2003.11.018 15246641

[B11] Kuijpers D Janssen CH van Dijkman PR Oudkerk M Dobutamine stress MRI. Part I. Safety and feasibility of dobutamine cardiovascular magnetic resonance in patients suspected of myocardial ischemia Eur Radiol 2004 14 10 1823 8 10.1007/s00330-004-2425-y 15278415

[B12] Hamilton CA Link KM Salido TB Epstein FH Hundley WG Is imaging at intermediate doses necessary during dobutamine stress magnetic resonance imaging? J Cardiovasc Magn Reson 2001 3 4 297 302 10.1081/JCMR-100108582 11777220

[B13] Rodgers GP Ayanian JZ Balady G Beasley JW Brown KA Gervino EV Paridon S Quinones M Schlant RC Winters WL Jr Achord JL Boone AW Hirshfeld JW Jr Lorell BH Rodgers GP Tracy CM Weitz HH American College of Cardiology/American Heart Association Clinical Competence Statement on Stress Testing. A Report of the American College of Cardiology/American Heart Association/American College of Physicians-American Society of Internal Medicine Task Force on Clinical Competence Circulation 2000 102 14 1726 38 11015355 10.1161/01.cir.102.14.1726

[B14] Darty SN Thomas MS Neagle CM Link KM Wesley-Farrington D Hundley WG Cardiovascular magnetic resonance imaging Am J Nurs 2002 102 12 34 8; quiz 12473928 10.1097/00000446-200212000-00013

[B15] Armstrong WF Pellikka PA Ryan T Crouse L Zoghbi WA Stress echocardiography: Recommendations for performance and interpretation of stress echocardiography. Stress Echocardiography Task Force of the Nomenclature and Standards Committee of the American Society of Echocardiography J Am Soc Echocardiogr 1998 11 1 97 104 10.1016/S0894-7317(98)70132-4 9487482

[B16] Cerqueira D Weisman NJ Dilsizian V Standardized myocardial segmentation and nomenclature for tomographic imaging of the heart Circulation 2002 105 539 542 10.1161/hc0402.102975 11815441

[B17] Hundley WG Hamilton CA Rerkpattanapipat P Magnetic resonance imaging assessment of cardiac function Curr Cardiol Rep 2003 5 1 69 74 10.1007/s11886-003-0040-1 12493163

[B18] Kaufman L Crooks L Sheldon P Hricak H Herfkens R Bank W The potential impact of nuclear magnetic resonance imaging on cardiovascular diagnosis Circulation 1983 67 2 251 7 6848214 10.1161/01.cir.67.2.251

[B19] Shan K Constantine G Sivananthan M Flamm SD Role of cardiac magnetic resonance imaging in the assessment of myocardial viability Circulation 2004 109 11 1328 34 10.1161/01.CIR.0000120294.67948.E3 15037539

[B20] Reeder SB Du YP Lima JA Bluemke DA Advanced cardiac MR imaging of ischemic heart disease Radiographics 2001 21 4 1047 74 11452080 10.1148/radiographics.21.4.g01jl281047

[B21] van der Wall EE Vliegen HW de Roos A Bruschke AV Magnetic resonance imaging in coronary artery disease Circulation 1995 92 9 2723 39 7586378 10.1161/01.cir.92.9.2723

[B22] Darty SN O'Neal J Wesley-Farrington D Davis AD Link KM Hundley G Cardiovascular magnetic resonance imaging Prog Cardiovasc Nurs 2004 19 2 60 7 10.1111/j.0889-7204.2004.02446.x 15133380

[B23] Vogel M Stern H Bauer R Bühlmeyer K Comparison of magnetic resonance imaging with cross-sectional echocardiography in the assessment of left ventricular mass in children without heart disease and in aortic isthmic coarctation Am J Cardiol 1992 69 9 941 4 10.1016/0002-9149(92)90797-3 1550025

[B24] Pennell DJ Underwood SR Manzara CC Swanton RH Walker JM Ell PJ Longmore DB Magnetic resonance imaging during dobutamine stress in coronary artery disease Am J Cardiol 1992 70 1 34 40 10.1016/0002-9149(92)91386-I 1615867

[B25] Thiele H Nagel E Paetsch I Schnackenburg B Bornstedt A Kouwenhoven M Wahl A Schuler G Fleck E Functional cardiac MR imaging with steady-state free precession (SSFP) significantly improves endocardial border delineation without contrast agents J Magn Reson Imaging 2001 14 4 362 7 10.1002/jmri.1195 11599059

[B26] Barkhausen J Ruehm SG Goyen M Buck T Laub G Debatin JF MR evaluation of ventricular function: True fast imaging with steady-state precession versus fast low-angle shot cine MR imaging: feasibility study Radiology 2001 219 1 264 9 11274568 10.1148/radiology.219.1.r01ap12264

[B27] Paetsch I Jahnke C Wahl A Gebker R Neuss M Fleck E Nagel E Comparison of dobutamine stress magnetic resonance, adenosine stress magnetic resonance, and adenosine stress magnetic resonance perfusion Circulation 2004 110 7 835 42 10.1161/01.CIR.0000138927.00357.FB 15289384

[B28] Schalla S Klein C Paetsch I Lehmkuhl H Bornstedt A Schnackenburg B Fleck E Nagel E Real-time MR image acquisition during high-dose dobutamine hydrochloride stress for detecting left ventricular wall-motion abnormalities in patients with coronary arterial disease Radiology 2002 224 3 845 51 10.1148/radiol.2243010945 12202724

[B29] Tsao J Boesiger P Pruessmann KP k-t BLAST and k-t SENSE: dynamic MRI with high frame rate exploiting spatiotemporal correlations Magn Reson Med 2003 50 5 1031 42 10.1002/mrm.10611 14587014

[B30] Jahnke C Paetsch I Gebker R Bornstedt A Fleck E Nagel E Accelerated 4 D dobutamine stress MR imaging with k-t BLAST: Feasibility and diagnostic performance Radiology 2006 241 3 718 28 Epub 2006 Oct 25 10.1148/radiol.2413051522 17065561

[B31] Kuijpers D Ho KY van Dijkman PR Vliegenthart R Oudkerk M Dobutamine cardiovascular magnetic resonance for the detection of myocardial ischemia with the use of myocardial tagging Circulation 2003 107 12 1592 7 10.1161/01.CIR.0000060544.41744.7C 12668491

[B32] Korosoglou G Lehrke S Wochele A Hoerig B Lossnitzer D Steen H Giannitsis E Osman NF Katus HA Strain-encoded CMR for the detection of inducible ischemia during intermediate stress JACC Cardiovasc Imaging 2010 3 4 361 71 10.1016/j.jcmg.2009.11.015 20394897

[B33] Paetsch I Föll D Kaluza A Luechinger R Stuber M Bornstedt A Wahl A Fleck E Nagel E Magnetic resonance stress tagging in ischemic heart disease Am J Physiol Heart Circ Physiol 2005 288 6 H2708 14 10.1152/ajpheart.01017.2003 15665054

[B34] Kraitchman DL Sampath S Castillo E Derbyshire JA Boston RC Bluemke DA Gerber BL Prince JL Osman NF Quantitative ischemia detection during cardiac magnetic resonance stress testing by use of FastHARP Circulation 2003 107 15 2025 30 10.1161/01.CIR.0000062684.47526.47 12668517

[B35] Kelle S Hamdan A Schnackenburg B Köhler U Klein C Nagel E Fleck E Prognostic value of negative dobutamine-stress cardiac magnetic resonance imaging Med Sci Monit 2009 15 10 MT131 136 19789518

[B36] Kelle S Hamdan A Schnackenburg B Köhler U Klein C Nagel E Fleck E Dobutamine stress cardiovascular magnetic resonance at 3 Tesla J Cardiovasc Magn Reson 2008 10 44 10.1186/1532-429X-10-44 18844984 PMC2572055

[B37] Baer FM Theissen P Smolarz K Voth E Sechtem U Schicha H Hilger HH Dobutamine versus dipyridamole magnetic resonance tomography: Safety and sensitivity in the detection of coronary stenoses Z Kardiol 1993 82 8 494 503 8212783

[B38] van Rugge FP van der Wall EE de Roos A Bruschke AV Dobutamine stress magnetic resonance imaging for detection of coronary artery disease J Am Coll Cardiol 1993 22 2 431 9 10.1016/0735-1097(93)90047-5 8335812

[B39] van Rugge FP van der Wall EE Spanjersberg SJ de Roos A Matheijssen NA Zwinderman AH van Dijkman PR Reiber JH Bruschke AV Magnetic resonance imaging during dobutamine stress for detection and localization of coronary artery disease. Quantitative wall motion analysis using a modification of the centerline method Circulation 1994 90 1 127 38 8025988 10.1161/01.cir.90.1.127

[B40] Nagel E Lehmkuhl HB Bocksch W Klein C Vogel U Frantz E Ellmer A Dreysse S Fleck E Noninvasive diagnosis of ischemia-induced wall motion abnormalities with the use of high-dose dobutamine stress MRI: Comparison with dobutamine stress echocardiography Circulation 1999 99 6 730 2 9989961 10.1161/01.cir.99.6.763

[B41] Hundley WG Hamilton CA Thomas MS Herrington DM Salido TB Kitzman DW Little WC Link KM Utility of fast cine magnetic resonance imaging and display for the detection of myocardial ischemia in patients not well suited for second harmonic stress echocardiography Circulation 1999 100 16 1697 702 10525488 10.1161/01.cir.100.16.1697

[B42] Wahl A Paetsch I Roethemeyer S Klein C Fleck E Nagel E High-dose dobutamine atropine stress cardiovascular MR imaging after coronary revascularization in patients with wall motion abnormalities at rest Radiology 2004 233 1 210 6 10.1148/radiol.2331030463 15304662

[B43] Nandalur KR Dwamena BA Choudhri AF Nandalur MR Carlos RC Diagnostic performance of stress cardiac magnetic resonance imaging in the detection of coronary artery disease: A meta-analysis J Am Coll Cardiol 2007 50 14 1343 53 10.1016/j.jacc.2007.06.030 17903634

[B44] Syed MA Paterson DI Ingkanisorn WP Rhoads KL Hill J Cannon RO Arai AE Reproducibility and inter-observer variability of dobutamine stress CMR in patients with severe coronary disease: Implications for clinical research J Cardiovasc Magn Reson 2005 7 5 763 8 10.1080/10976640500287414 16353436

[B45] Paetsch I Jahnke C Ferrari VA Rademakers FE Pellikka PA Hundley WG Poldermans D Bax JJ Wegscheider K Fleck E Nagel E Determination of interobserver variability for identifying inducible left ventricular wall motion abnormalities during dobutamine stress magnetic resonance imaging Eur Heart J 2006 27 12 1459 64 10.1093/eurheartj/ehi883 16613929

[B46] Lubbers DD Janssen CH Kuijpers D van Dijkman PR Overbosch J Willems TP Oudkerk M The additional value of first pass myocardial perfusion imaging during peak dose of dobutamine stress cardiac MRI for the detection of myocardial ischemia Int J Cardiovasc Imaging 2008 24 1 69 76 10.1007/s10554-006-9205-5 17566871 PMC2121120

[B47] Gebker R Jahnke C Manka R Hamdan A Schnackenburg B Fleck E Paetsch I Additional value of myocardial perfusion imaging during dobutamine stress magnetic resonance for the assessment of coronary artery disease Circ Cardiovasc Imaging 2008 1 2 122 30 Epub 2008 Jul 30 10.1161/CIRCIMAGING.108.779108 19808529

[B48] Gebker R Mirelis JG Jahnke C Hucko T Manka R Hamdan A Schnackenburg B Fleck E Paetsch I Influence of left ventricular hypertrophy and geometry on diagnostic accuracy of wall motion and perfusion magnetic resonance imaging during dobutamine stress Circ Cardiovasc Imaging 2010 in press 20576810 10.1161/CIRCIMAGING.109.923672

[B49] Jekic M Foster EL Ballinger MR Raman SV Simonetti OP Cardiac function and myocardial perfusion immediately following maximal treadmill exercise inside the MRI room J Cardiovasc Magn Reson 2008 10 1 3 10.1186/1532-429X-10-3 18272005 PMC2244608

[B50] Rerkpattanapipat P Morgan TM Neagle CM Link KM Hamilton CA Hundley WG Assessment of preoperative cardiac risk with magnetic resonance imaging Am J Cardiol 2002 90 4 416 9 10.1016/S0002-9149(02)02501-8 12161234

[B51] Hundley WG Morgan TM Neagle CM Hamilton CA Rerkpattanapipat P Link KM Magnetic resonance imaging determination of cardiac prognosis Circulation 2002 106 18 2328 33 10.1161/01.CIR.0000036017.46437.02 12403662

[B52] Hundley WG Rerkpattanapipat P Little WC Link KM Morgan TM Relation of cardiac prognosis to segment location with apical left ventricular ischemia Am J Cardiol 2003 92 10 1206 8 10.1016/j.amjcard.2003.07.033 14609599

[B53] Kuijpers D van Dijkman PR Janssen CH Vliegenthart R Zijlstra F Oudkerk M Dobutamine stress MRI. Part II. Risk stratification with dobutamine cardiovascular magnetic resonance in patients suspected of myocardial ischemia Eur Radiol 2004 14 11 2046 52 10.1007/s00330-004-2426-x 15278416

[B54] Jahnke C Nagel E Gebker R Kokocinski T Kelle S Manka R Fleck E Paetsch I Prognostic value of cardiac magnetic resonance stress tests: adenosine stress perfusion and dobutamine stress wall motion imaging Circulation 2007 115 13 1769 76 Epub 2007 Mar 12 10.1161/CIRCULATIONAHA.106.652016 17353441

[B55] Sketch MH Mohiuddin SM Lynch JD Zencka AE Runco V Significant sex differences in the correlation of electrocardiographic exercise testing and coronary arteriograms Am J Cardiol 1975 36 2 169 73 10.1016/0002-9149(75)90521-4 1155337

[B56] Mieres JH Shaw LJ Arai A Budoff MJ Flamm SD Hundley WG Marwick TH Mosca L Patel AR Quinones MA Redberg RF Taubert KA Taylor AJ Thomas GS Wenger NK Cardiac Imaging Committee, Council on Clinical Cardiology, and the Cardiovascular Imaging and Intervention Committee, Council on Cardiovascular Radiology and Intervention, American Heart Association Role of noninvasive testing in the clinical evaluation of women with suspected coronary artery disease: Consensus statement from the Cardiac Imaging Committee, Council on Clinical Cardiology, and the Cardiovascular Imaging and Intervention Committee, Council on Cardiovascular Radiology and Intervention, American Heart Association Circulation 2005 111 5 682 96 10.1161/01.CIR.0000155233.67287.60 15687114

[B57] Shaw LJ Peterson ED Kesler K Hasselblad V Califf RM A meta analysis of predischarge risk stratification after acute myocardial infarction with stress electrocardiographic, myocardial perfusion, and ventricular function imaging Am J Cardiol 1996 78 12 1327 37 10.1016/S0002-9149(96)00653-4 8970402

[B58] Gebker R Jahnke C Hucko T Manka R Mirelis JG Hamdan A Schnackenburg B Fleck E Paetsch I Dobutamine stress magnetic resonance imaging for the detection of coronary artery disease in women Heart 2010 96 8 616 20 10.1136/hrt.2009.175521 19687013

[B59] Wallace EL Morgan TM Walsh TF Dall'Armellina E Ntim W Hamilton CA Hundley WG Dobutamine cardiac magnetic resonance results predict cardiac prognosis in women with known or suspected ischemic heart disease J Am Coll Cardiol Cardiovasc Imaging 2009 2 3 299 307 10.1016/j.jcmg.2008.10.015 PMC2920607 19356575

[B60] Dall'Armellina E Morgan TM Mandapaka S Ntim W Carr JJ Hamilton CA Hoyle J Clark H Clark P Link KM Case D Hundley WG Prediction of cardiac events in patients with reduced left ventricular ejection fraction with dobutamine cardiovascular magnetic resonance assessment of wall motion score index J Am Coll Cardiol 2008 52 4 279 86 10.1016/j.jacc.2008.04.025 18634983 PMC3666037

[B61] Walsh TF Dall'Armellina E Chughtai H Morgan TM Ntim W Link KM Hamilton CA Kitzman DW Hundley WG Adverse effect of increased left ventricular wall thickness on five year outcomes of patients with negative dobutamine stress J Cardiovasc Magn Reson 2009 11 1 25 10.1186/1532-429X-11-25 19650895 PMC2730053

[B62] Charoenpanichkit C Morgan TM Hamilton CA Wallace EL Robinson K Ntim WO Hundley WG Left ventricular hypertrophy influences cardiac prognosis in patients undergoing dobutamine cardiac stress testing Circ Cardiovasc Imaging 2010 3 4 392 7 10.1161/CIRCIMAGING.109.912071 20442370 PMC3053573

[B63] Korosoglou G Elhmidi Y Steen H Schellberg D Riedle N Ahrens J Lehrke S Merten C Radeleff J Zugck C Giannitsis E Katus HA Prognostic value of high-dose dobutamine stress magnetic resonance imaging in 1493 consecutive patients: Assessment of myocardial wall motion and perfusion J Am Cardiol Coll 2010 56 1225 34 10.1016/j.jacc.2010.06.020 20883929

[B64] Cigarroa CG deFilippi CR Brickner ME Alvarez LG Wait MA Grayburn PA Dobutamine stress echocardiography identifies hibernating myocardium and predicts recovery of left ventricular function after coronary revascularization Circulation 1993 88 2 430 6 8339406 10.1161/01.cir.88.2.430

[B65] Baer FM Voth E Schneider CA Theissen P Schicha H Sechtem U Comparison of low-dose dobutamine-gradient-echo magnetic resonance imaging and positron emission tomography with [18F] fluorodeoxyglucose in patients with chronic coronary artery disease: A functional and morphological approach to the detection of residual myocardial viability Circulation 1995 91 4 1006 15 7850935 10.1161/01.cir.91.4.1006

[B66] Baer FM Theissen P Schneider CA Voth E Sechtem U Schicha H Erdmann E Dobutamine magnetic resonance imaging predicts contractile recovery of chronically dysfunctional myocardium after successful revascularization J Am Coll Cardiol 1998 31 5 1040 8 10.1016/S0735-1097(98)00032-1 9562005

[B67] Sandstede JJ Bertsch G Beer M Kenn W Werner E Pabst T Lipke C Kretschmer S Neubauer S Hahn D Detection of myocardial viability by low-dose dobutamine Cine MR imaging Magn Reson Imaging 1999 17 10 1437 43 10.1016/S0730-725X(99)00095-8 10609992

[B68] Geskin G Kramer CM Rogers WJ Theobald TM Pakstis D Hu YL Reichek N Quantitative assessment of myocardial viability after infarction by dobutamine magnetic resonance tagging Circulation 1998 98 3 217 23 9697821 10.1161/01.cir.98.3.217

[B69] Bogaert J Maes A Van de Werf F Bosmans H Herregods MC Nuyts J Desmet W Mortelmans L Marchal G Rademakers FE Functional recovery of subepicardial myocardial tissue in transmural myocardial infarction after successful reperfusion: An important contribution to the improvement of regional and global left ventricular function Circulation 1999 99 1 36 43 9884377 10.1161/01.cir.99.1.36

[B70] Sayad DE Willett DL Hundley WG Grayburn PA Peshock RM Dobutamine magnetic resonance imaging with myocardial tagging quantitatively predicts improvement in regional function after revascularization Am J Cardiol 1998 82 9 1149 51, A10 10.1016/S0002-9149(98)00579-7 9817504

[B71] Dendale PA Franken PR Waldman GJ De Moor DG Tombeur DA Block PF De Roos A Low-dosage dobutamine magnetic resonance imaging as an alternative to echocardiography in the detection of viable myocardium after acute infarction Am Heart J 1995 130 1 134 40 10.1016/0002-8703(95)90248-1 7611103

[B72] Saito I Watanabe S Masuda Y Detection of viable myocardium by dobutamine stress tagging magnetic resonance imaging with three-dimensional analysis by automatic trace method Jpn Circ J 2000 64 7 487 94 10.1253/jcj.64.487 10929775

[B73] Kramer CM Malkowski MJ Mankad S Theobald TM Pakstis DL Rogers WJ Jr Magnetic resonance tagging and echocardiographic response to dobutamine and functional improvement after reperfused myocardial infarction Am Heart J 2002 143 6 1046 51 10.1067/mhj.2002.122515 12075262

[B74] Zamorano J Delgado J Almería C Moreno R Gómez Sánchez M Rodrigo J Fernández C Ferreiros J Rufilanchas J Sánchez-Harguindey L Reason for discrepancies in identifying myocardial viability by thallium-201 redistribution, magnetic resonance imaging, and dobutamine echocardiography Am J Cardiol 2002 90 5 455 9 10.1016/S0002-9149(02)02513-4 12208401

[B75] Kramer CM Rogers WJ Jr Mankad S Theobald TM Pakstis DL Hu YL Contractile reserve and contrast uptake pattern by magnetic resonance imaging and functional recovery after reperfused myocardial infarction J Am Coll Cardiol 2000 36 6 1835 40 10.1016/S0735-1097(00)00945-1 11092653

[B76] Lauerma K Niemi P Hänninen H Janatuinen T Voipio-Pulkki LM Knuuti J Toivonen L Mäkelä T Mäkijärvi MA Aronen HJ Multimodality MR imaging assessment of myocardial viability: combination of first-pass and late contrast enhancement to wall motion dynamics and comparison with FDG PET-initial experience Radiology 2000 217 3 729 36 11110935 10.1148/radiology.217.3.r00dc18729

[B77] Wellnhofer E Olariu A Klein C Gräfe M Wahl A Fleck E Nagel E Magnetic resonance low-dose dobutamine test is superior to SCAR quantification for the prediction of functional recovery Circulation 2004 109 18 2172 4 10.1161/01.CIR.0000128862.34201.74 15117834

[B78] Bove CM DiMaria JM Voros S Conaway MR Kramer CM Dobutamine response and myocardial infarct transmurality: Functional improvement after coronary artery bypass grafting--initial experience Radiology 2006 240 3 835 41 10.1148/radiol.2403051150 16926330

[B79] Motoyasu M Sakuma H Ichikawa Y Ishida N Uemura S Okinaka T Isaka N Takeda K Nakano T Prediction of regional functional recovery after acute myocardial infarction with low dose dobutamine stress cine MR imaging and contrast enhanced MR imaging J Cardiovasc Magn Reson 2003 5 4 563 74 10.1081/JCMR-120025233 14664134

[B80] Kaandorp TA Bax JJ Schuijf JD Viergever EP van Der Wall EE de Roos A Lamb HJ Head-to-head comparison between contrast-enhanced magnetic resonance imaging and dobutamine magnetic resonance imaging in men with ischemic cardiomyopathy Am J Cardiol 2004 93 12 1461 4 10.1016/j.amjcard.2004.03.003 15194013

[B81] Kaandorp TA Lamb HJ Bax JJ Boersma E Viergever EP van der Wall EE de Roos A Prediction of beneficial effect of beta blocker treatment in severe ischaemic cardiomyopathy: assessment of global left ventricular ejection fraction using dobutamine stress cardiovascular magnetic resonance Heart 2005 91 11 1471 2 10.1136/hrt.2004.051425 16230448 PMC1769186

